# Combinations of Parent-Related Risk Factors Explaining Family Violence Toward Children and Spouse

**DOI:** 10.1177/08862605231208421

**Published:** 2023-11-08

**Authors:** Tuija Leppäkoski, Maaret Vuorenmaa, Eija Paavilainen

**Affiliations:** 1 Tampere University, Finland; 2 Finnish Institute for Health and Welfare (THL), City of Helsinki, Finland; 3 South Ostrobothnia Hospital District, City of Seinäjoki, Finland

**Keywords:** prevention of child abuse, child abuse, children exposed to domestic violence, domestic violence, neglect, physical abuse, treatment/intervention

## Abstract

The purpose of the study was to investigate parent-related risk factor combinations that explain family violence (FV), which refers to intimate partner violence and child maltreatment (CM). The data were collected from parents with a 4-year-old child using a nationwide retrospective cross-sectional survey conducted in Finland (FinChildren) (*N* = 10,737). The research questions were as follows: (a) How are parent-related risk factors associated with FV against children and the spouse? (b) How does the accumulation of parent-related risk factors within three risk factor clusters explain FV? Analyses were carried out using cross-tabulations with χ^2^ tests, an exploratory factor analysis (EFA), and binary logistic regression analyses. The risk factor clusters built based on the EFA were as follows: parental well-being, parent’s childhood adversities, and parent’s health. Our results indicated that even a single risk factor predicted the likelihood of FV. In the well-being risk factor cluster, the odds for the occurrence of FV in parents with one well-being risk factor were double (odds ratios [OR] = 2.21; confidence intervals [CI]: [1.99, 2.45]) and in parents with at least four risk factors was six times (OR = 6.05; CI: [4.48, 8.18]) compared to those with no risk factor. We concluded that (a) the more different risk factors parents had, the more likely they were to report FV and (b) the accumulation of risk factors for well-being contributes most to the occurrence of the risk of FV. As a result, we emphasize the importance of identifying families with concurrent risk factors. However, any individual concerns must be addressed with parents and they must be supported in coping with their everyday life.

## Introduction

Family violence (FV) is a global health problem with direct and indirect consequences on long-term economic and welfare costs (Bellis et al., 2019; Mo et al., 2020; Peterson et al., 2018). The concept of FV is understood rather similarly across international research literature, although cultural issues associated with FV and the definitions of FV vary between countries and studies. In this study based in Finland, FV is considered to include both child maltreatment (CM) and intimate partner violence (IPV), sometimes also referred to as domestic violence (DV) (Leppäkoski et al., 2021). In the present study, CM is considered to comprise psychological and physical abuse, including corporal punishment or parental child neglect, while IPV/CV includes psychological, physical, sexual, and/or financial violence in the parents’ current or former intimate relationship.

Research has shown that the prevalence of CM varies from study to study, depending on, for example, data collection methods. According to a systematic literature review study using the self-reporting of maltreatment (Moody et al., 2018), in Europe, the rates of lifetime physical abuse were 12.0% (6.9%–23.0%) and 27.0% (7.0%–43.0%) for girls and boys, respectively. The results of another study based on large nationwide survey data in Finland showed that, of 4-year-olds, 44% had experienced at least one form of psychological abuse and 14% physical abuse in the last 12 months as reported by their parents. Furthermore, these forms of violence co-occurred in 25% of reported cases (Leppäkoski et al., 2021).

Based on growing international research evidence, CM and IPV may co-occur, increasing the risk of children’s adverse health outcomes (Brown et al., 2021). This emphasizes the importance of prevention. A review by Birarra et al. (2016) showed that the prevalence of the co-occurrence of IPV with different types of CM ranged from 12% to 70%. In a study conducted in the Netherlands, the prevalence of CM was 46% in families with DV based on, for example, observations made by professionals working with children (van Berkel et al., 2020). A most recent Finnish study (Leppäkoski et al., 2021) also showed that IPV was the risk factor most likely to predict an increased risk for both psychological abuse (OR 4.01) and physical abuse (OR 2.19) of a child. Moreover, repeated violence against children is particularly harmful to the health of the child (Finkelhor et al., 2009). In Finland, according to the Statistics on offences and coercive measures (2021), 2% of underaged children had been victims for several years in the period 2009–2020.

The victims and perpetrators of IPV include both men and women (Bates, 2016; Costa et al., 2015). For example, in a study by Costa et al. (2015), male and female victims of physical assault, respectively, ranged from 9.7% and 8.5% to 31.2% and 23.1%. According to the 2019 Finnish National Crime Victims Survey (Danielsson & Näsi, 2020), 7.0% of women and 3.2% of men (15–74 years) had experienced violence (all acts of violence combined) committed by their former or current partner.

However, it should be noted that the actual occurrence of FV is likely to be underestimated: not all cases are reported to the police, as a result of which the actual number may be higher than reported. Therefore, the professionals working with families play an important role in identifying and responding to FV. As a result, the purpose of this study was to investigate whether some parents’ characteristics may be risk factors for predicting the occurrence of FV.

### Parents’ Characteristics Associated with FV

According to several studies, parents’ experiences of maltreatment in their own childhood as an individual risk factor—also referred to as intergenerational transmission—may be linked to an increased risk of CM (Bartlett et al., 2017; Liel et al., 2020; Peltonen et al., 2014). Bartlett et al. (2017) showed that both a maternal history of substantiated neglect and multiple types of maltreatment (neglect and physical or sexual abuse) were associated with an increased risk of CM. Peltonen et al. (2014) indicated that corporal punishment experienced by the mother in her childhood or used by the mother as a discipline technique strongly increased (OR 11.14) the likelihood of severe acts of violence.

Moreover, young children living in an environment with IPV may also be exposed to harsh parenting styles (i.e., harsh punishment) (Grasso et al., 2016; Liel et al., 2020). For example, the mothers reporting a greater occurrence of IPV in the form of physical assault more often engage in mild to more severe forms of disciplinary violence that may harm their child (ORs = 3.8–5.0) (Grasso et al., 2016). A study using two separate representative samples of Finnish and Swedish parents of children between 0 and 12 years of age showed that the use of any corporal punishment as a method of discipline by the father strongly increased (OR 8.17) the likelihood of severe violent acts (Ellonen et al., 2017). A national survey conducted in Finland showed that parental approval of corporal punishment, such as pulling the child’s hair, was most likely to predict an increased risk of physical abuse (OR 13.7) (Leppäkoki et al., 2021). According to Younas and Morrison Gutman (2022), corporal punishment was also among the specific risk factors for child physical abuse.

External factors have also been found to be associated with CM. Doidge et al. (2017) indicated that higher levels of economic disadvantage, poor parental mental health and substance use, and parents’ social instability were strongly associated with an increased risk of CM. Furthermore, a parental history of antisocial behavior or criminal offending, a history of mental or psychiatric problems, and a combination of mental issues with experiences of CM in own childhood were identified as risk factors for child neglect by Mulder et al. (2018). In addition, work- or family-related stress and a lack of help in dealing with parenting problems were detected as risk factors for severe acts of violence toward one’s own child (Peltonen et al., 2014). Meanwhile, Van Berkel et al. (2020) showed that the most important risk factors for CM were low parental education, parental employment, immigrant status, and single parenthood. Liel et al. (2020) also identified the child’s age and parental stress as significant risk factors (*p* < .05) for child abuse in addition to IPV. Neglect was also found to be associated with distress in the parents’ relationship, adverse childhood experiences (here: a history of CM in the parent’s childhood), young maternal age, cramped housing, and a history of migration.

There is strong evidence of associations between adverse childhood experiences such as FV and poor health later in life (Bellis et al., 2019; Chartier et al., 2010; Hughes et al., 2017; Mosley-Johnson et al., 2019). A Canadian study (Chartier et al., 2010) showed that, in a representative population sample, 72% of respondents reported at least one adverse childhood experience (childhood physical and sexual abuse, parental marital conflict, poor parent–child relationship, low parental education, and parental mental health) and 37% two or more of such experiences. The study concluded that the accumulation of these adverse experiences increases the risk of poor health in adulthood. Similarly, in a longitudinal cohort study conducted in the United States (Mosley-Johnson et al., 2019), some adverse childhood experiences, such as abuse and household dysfunction were significantly associated with lower satisfaction with life, lower psychological well-being, and lower social well-being. According to a systematic review and meta-analysis (Hughes et al., 2017), a history of multiple adverse childhood experiences (ACEs) is a major risk factor for many health conditions. The outcomes most strongly associated with multiple ACEs increase the risk of ACEs for the next generation (e.g., violence, mental illness, and substance use). This link between ACEs and later health problems makes it important to focus on prevention.

Today, it is increasingly understood that many risks of CM can occur simultaneously and may be cumulative in nature (Patwardhan et al., 2017; Wallander et al., 2019; Yang & Maguire-Jack, 2018). In other words, the more risk factors a child and/or family are facing, the more vulnerable the child is to violence. Patwardhan et al. (2017) indicated that the most prominent family risks contributing to the cumulative risk scale were socioeconomic disadvantage (e.g., income, unemployment, housing instability) and parental characteristics (e.g., mental/physical health, parents’ use of alcohol, IPV). For example, Wallander et al. (2019) highlighted that the accumulation of exposure to multiple risk factors is more harmful to a child than individual risk factor exposure. Yang and Maguire-Jack (2018) concluded that beyond a certain number of risks (5 or more), families may not be able to cope adequately with the stress they are under, which increases the likelihood of CM. Therefore, enabling CM prevention requires simultaneously and systematically addressing a diverse set of risk factors, also including any worries expressed by parents rather than only focusing on a particular issue (Lepistö et al., 2022). There is also a need for interventions that aim at helping parents, such as discussions (Bekaert et al., 2021; Lepistö et al., 2022).

Based on the previous research evidence, it can be concluded that CM and IPV may co-occur and involve many family- and parent-related background factors (e.g., Liel et al., 2020). Furthermore, FV has many harmful consequences on the well-being of affected families. Professionals working with families with children play an essential role in identifying those families that may have an increased risk of FV.

The purpose of this study was to investigate, based on survey data, what kinds of parents have reported FV, that is, parents who have reported experiences of IPV and/or CM, and what kinds of combinations of the parent-related factors there are. The produced knowledge will enable professionals working with families with children to understand potential connections between risk factors and help them ask suitable questions from parents (Rantanen et al., 2022). The objective is that professionals can find ways to help families sufficiently early and increase their well-being. As the consequences of CM affect an individual’s entire life span, we wish to put emphasize on the importance of intervening in problems in families as early as possible. To promote this aim, the following questions were formed: (a) How are parent-related risk factors associated with FV against children and spouse? (b) How does the accumulation of parent-related risk factors within the risk factor clusters explain FV?

## Methods

This study is a nationwide retrospective cross-sectional survey (FinChildren) conducted by the Finnish Institute for Health and Welfare (THL).

### Setting and Participants

The participants were parents of a 4-year-old child in Finland. The target group included all families whose 4-year-old child underwent an extensive health examination at a child health clinic in 2018 in the municipalities participating in the data collection. In total, 290 out of 295 municipalities in mainland Finland participated in the data collection. Public health nurses served as contact persons in the municipalities and provided questionnaires to the children’s parents. Overall, 17,009 families gave their consent to participate in the study. For 8,720 children, one or both parents filled out the questionnaire (24%). The total number of responses by parents was 10,737 (Vuorenmaa, 2019).

### Ethical Considerations

The study adhered to the guidelines for good scientific practice published by the Finnish National Advisory Board on Research Integrity (2012) (https://www.tenk.fi/en) and the Declaration of Helsinki (2008) (World Medical Association, 2022). The municipalities were requested to provide a research permit prior to the data collection process. The families were informed about the study orally and in writing. Study participation was voluntary and the parents could choose to withdraw at any point. The child’s official guardian signed a written consent form for participation in the study at a child health clinic. The study was approved by the Ethical Committee of the Finnish Institute for Health and Welfare (773/2017).

### Measures

Our research data were based on a dataset collected through the 2018 Children’s Health, Well-being and Services survey (FinChildren). The survey asked the parents about issues such as welfare, health and functional capacity, lifestyle, the safety of the growth environment, and the need, availability, and adequacy of services and support (Finnish Institute of Health and Welfare, 2018). We selected certain variables for closer examination based on the aim of our study:

#### Background Variables

The examined background variables included the parents’ gender (female/male), age (<35 years old/≥35 years old), the number of children (the 4-year-old is the only child/there are other children in the family), and the number of adults (one adult in the family/two adults in the family).

#### Parent-Related Risk Factors for FV

The parent-related risk factors for FV examined in this study were parents’ chronic illness or other chronic health problems (yes/no), self-rated health at most on an average level (yes = average, fairly bad or very bad/no = very good or fairly good), restrictions caused by a health problem (yes = severely restricted or restricted, but not severely/no = not restricted at all), feelings of loneliness (yes = fairly often or all the time/no = sometimes, very rarely or never), depressive symptoms, psychological distress, experiences with parenting, and the parents’ own childhood adversities.

##### Depressive Symptoms

Depressive symptoms were measured using the Patient Health Questionnaire 2 (PHQ-2) (Kroenke et al., 2003). The parents were asked the following questions: “Over the past 12 months, have you ever had a period of 2 weeks or more when for most of the time you have felt (a) down, melancholic, or depressed, (b) have lost your interest in most things that usually give you pleasure?”. The response options were “yes” and “no.” Parents had depressive symptoms if they answered “yes” to at least one item on the instrument.

##### Psychological Distress

Psychological distress was measured using the Mental Health Inventory (MHI-5) (Cuijpers et al., 2009). The parents were asked the following question: “Over the past 4 weeks, for how much of the time have you. . ..?” and given the following statements: (a) felt very nervous, (b) had such a low mood that nothing could cheer me up, (c) felt calm and peaceful, (d) felt downhearted and sad, and (e) felt happy. The response options were “all the time,” “most of the time,” “a good bit of the time,” “some time,” “a little of the time,” and “none of the time.” The scores for items 3 and 5 were reversed, after which the total scores for the sections were summed up (sum 5–30) and converted to a scale of 0 to 100. MHI-5 can be reported as a continuous variable or the score 52 can be used as the cut-off point, in which case those scoring 52 or below will have clinically significant psychological distress (e.g., Rumpf et al., 2001; Viertiö et al., 2017). In this study, MHI-5 was examined with a cutoff point that divided parents into significantly psychologically distressed (score ≤ 52) and not significantly distressed (score > 52) parents.

##### Parenting Experiences

Experiences with parenting were measured with two items. The parents were asked: “How often is the following true for you: (a) I feel inadequate as a parent and (b) I am worried about my coping as a parent.” The response options were “never,” “rarely,” “sometimes,” “often,” and “always.” The respondents were considered to feel inadequate as a parent and worried about their coping as a parent when they chose the response option “often” or “always.”

##### Childhood Adversities

Childhood adversities were measured using questions that began as follows: “If you think back to the years when you were growing up, that is, before the age of 16, did you experience. . .?” (Aromaa & Koskinen, 2002). The respondents were asked about seven types of adversity: long-term financial difficulties in the childhood family, parents’ regular unemployment, parents’ alcohol problems, parents’ mental health problems, serious conflicts within the family, parents’ divorce, and bullying at school. The response options were “yes,” “no,” and “I don’t know.” The occurrence of each adversity was coded as “yes” or “no” (the latter including both “no” and “I don’t know” responses).

#### Family Violence

FV included both violence experienced by the child (CM) as reported by parents, and IPV experienced by the parent(s). Six items were concerned with violence experienced by the child. The parents were asked whether they personally, the child’s other parent, or the parent’s spouse or former spouse had been violent toward the child in the previous 12 months. The forms of violence included were as follows: (a) throwing, hitting, or kicking an object in anger in front of the child, (b) leaving the child without care and attention for a longer period, (c) verbally threatening the child with violence, and (d) calling the child names, or belittling, severely criticizing or otherwise verbally abusing the child, (e) pinching, pulling the child’s hair or slapping the child, and (f) kicking or hitting the child. The response alternatives were “never,” “once,” “sometimes,” and “often.”

Four items we used to ask about IPV experienced by the parent(s). The parents were asked whether they had experienced any of the following in their intimate relationship (the parent’s current or former intimate relationship) in the previous 12 months: physical, emotional, sexual, or financial violence. The response alternatives were “yes” and “no.” The sum variable of FV was formed and coded as follows: the parent reported that the child had been exposed to at least one form of violence and/or the parent had been exposed to at least one form of IPV at least once in the previous 12 months (yes/no).

### Statistical Analysis

First, the data were described using frequencies and percentages. Second, associations between FV and background variables and parent-related risk factors were described using cross-tabulations with chi-squared tests of significance and with Cramer’s *V* tests of effect sizes. In our study, effect sizes directly reflect the strength of the relationship between variables (Barry et al., 2016; Sullivan & Feinn, 2012). Interpretations of effect sizes are related to the degrees of freedom (df). When *df* = 1, the effect size is classified as follows: small (*V* = .10), medium (*V* = .30), and large (*V* = .50). As the degrees of freedom increase, a smaller *V*-values means a larger effect size. When *df* = 3, effect size is classified as follows: small (*V* = .06), medium (*V* = .17), and large (*V* = .29) and when *df* = 4, the effect size is classified as follows: small (*V* = .05), medium (*V* = .15), and large (*V* = .25) (Hae-Young Kim, 2017).

The third phase involved performing an exploratory factor analysis (EFA). The broad aim of EFA is to summarize data so that relationships and patterns can be easily interpreted and understood. It can be used to regroup variables into a limited set of clusters based on shared variance (Yong & Pearce, 2013). In this study, the aim of the factor analysis was to determine whether parent-related risk factors are linked through some latent factors and whether risk factors could be lumped into “parent-related risk factor clusters” in this way. Only those risk factors that showed significant (*p* < .005) associations with FV were included in the EFA. Principal component analysis (PCA) with Varimax rotation was used as an extraction method. Other methods were also tested, but the factor structure remained substantially similar. Communalities >0.30 and factor loadings >0.50 were considered acceptable (Plichta & Kelvin, 2013). Next, based on the factors of the EFA, three sum variables were constructed as the risk factor clusters.

Finally, we explored how the accumulation of parent-related risk factors within the risk factor clusters explains FV. Both cross-tabulations with chi-square tests of significance and with Cramer’s *V*-tests of effect sizes and binary logistic regression analyses were used. Regression analyses were adjusted for parents’ gender, age, the number of children, and the number of adults in the family. Logistic regression is reported with odds ratios (ORs) and associated 95% confidence intervals (CI). The analyses were performed using IBM SPSS (Version 27 for Windows) (IBM Corp, Released, 2020). Statistical significance was set at ≤.05.

## Results

### Parent-Related Risk Factors Associated with FV Toward Children and Spouse

#### Parents’ Backgrounds

Of the parents (*N* = 10,737) responding to the survey, 71.6% were female and 28.4% were male. Just over half of the respondents (55.1%) were aged 35 or over. Most (83.8%) of the parents had other children in addition to the 4-year-old and (92.6%) lived with their spouse (Table 1).

**Table 1.** Parents’ Background Variables and Parent-Related Risk Factors for Family Violence (n, %).

	*n* (%)
All parents (*N* = 10,737)	
Parents’ background variables	
Gender (*n* = 10,699)	
Female	7,636 (71.6)
Male	3,033 (28.4)
Age (*n* = 10,697)	
<35 years old	4,802 (44.9)
≥35 years old	5,895 (55.1)
Number of children (*n* = 10,581)	
4-year-old is only child	1,716 (16.2)
Other children in the family	8,865 (83.8)
Number of adults (*n* = 10,668)	
One adult in the family	791 (7.4)
Two adults in the family	9,877 (92.6)
Parent-related risk factors for family violence	
Parent’s recent situation	
Has a chronic illness or other chronic health problem (*n* = 10,667)	
Yes	2,827 (26.5)
No	7,840 (73.5)
Perceived health condition at most average (*n* = 10,669)	
Yes	1,022 (9.6)
No	9,647 (90.4)
Has restrictions caused by a health problem (*n* = 10,652)	
Yes	1,865 (17.5)
No	8,787 (82.5)
Felt lonely fairly often or all the time (*n* = 10,696)	
Yes	729 (6.8)
No	9,967 (93.2)
Depressive symptoms for at least a period of 2 weeks over the past 12 months (*n* = 10,629)	
Yes	2,038 (19.2)
No	8,591 (80.8)
Psychological distress over the past 4 weeks (*n* = 10,537)	
Yes	649 (6.2)
No	9,888 (93.8)
Been worried about coping as a parent often or always (*n* = 10,687)	
Yes	687 (6.4)
No	10,000 (93.6)
Felt inadequate as a parent often or always (*n* = 10,663)	
Yes	1,275 (12.0)
No	9,388 (88.0)
Childhood adversities	
Long-term financial difficulties in the family (*n* = 10,669)	
Yes	3,796 (35.6)
No	6,873 (64.4)
Parents’ divorce (*n* = 10,668)	
Yes	2,616 (24.5)
No	8,052 (75.5)
Parents’ regular unemployment (*n* = 10,675)	
Yes	1,778 (16.7)
No	8,897 (83.3)
Bullying at school (*n* = 10,671)	
Yes	3,736 (35.0)
No	6,935 (65.0)
Serious conflicts within the family (*n* = 10,665)	
Yes	2,982 (28.0)
No	7,683 (72.0)
Parent’s mental health problems (*n* = 10,688)	
Yes	1,578 (14.8)
No	9,110 (85.2)
Parent’s alcohol problems (*n* = 10,689)	
Yes	2,641 (24.7)
No	8,048 (75.3)
Family violence reported by the parents	
Parents reported exposure to at least one form of IPV in the last 12 months at least once (*n* = 10,465)	
Yes	1,035 (9.9)
No	9,430 (90.1)
The parent’s 4-year-old child has experienced at least one form of CM in the last 12 months at least once as reported by parents (*n* = 10,689)	
Yes	4,640 (43.4)
No	6,049 (56.6)
The child has experienced at least one form of CM and/or parents have been exposed to at least one form of IPV in the last 12 months (*n* = 10,694)	
Yes	4,890 (45.7)
No	5,804 (54.3)

*Note.* CM = child maltreatment; IPV = intimate partner violence.

#### Parent-Related Risk Factors for FV

Table 1 shows the distributions of parent-related risk factors for FV chosen for this study. Of the parents of the 4-year-old children, 26.5% had reported a chronic illness or other health problem. Almost 10% of the parents (9.6%) had felt that their health was at best average, 17.5% had felt that their health problem had restricted their lives, and 19.2% had experienced depressive symptoms for a period of at least 2 weeks over the past 12 months. Twelve percent of the parents had often or always felt inadequate as a parent. Furthermore, the parents described their recent situation as follows: they had felt lonely fairly often or all the time (6.8%), experienced psychological distress over the past 4 weeks (6.2%), and had been worried about their coping as a parent often or always (6.4%) (Table 1).

In the area of childhood adversities, 35.6% of the parents reported having experienced long-term financial difficulties in their family, and almost as many had been subject to school bullying (35.0%). Almost a quarter of the mentions (24.5%) concerned parents’ divorce or alcohol problems. Serious intra-family conflicts were mentioned as childhood adversity in 28.0% of cases. The least often mentioned childhood adversities included the parents’ regular unemployment (16.7%) and parents’ mental health problems (14.8%) (Table 1).

#### FV Reported by the Parents

Almost 10% (9.9%) (1,035 out of 10,465) of the parents of 4-year-old children who responded to the survey reported having been exposed to at least one form of IPV in their current or former intimate relationship at least once in the last 12 months. Of all the parents, 43.4% (4,640 out of 10,465) reported that their 4-year-old child had been subjected to at least one form of violence, at least once in the last 12 months by the parent responding to the survey, the child’s other parent, or the parent’s spouse or former spouse. A total of 45.7% (4,890 out of 10,694) of the parents reported at least one form of IPV and/or CM. Meanwhile, 54.3% of the parents did not report any form of FV (Table 1).

### Associations Between FV and Background Variables and Parent-Related Risk Factors for Family Violence

Table 2 shows the associations between FV (parents who have reported IPV and/or CM) and the background variables and parent-related risk factors for FV. FV was reported more often by female than male respondents (47.7% vs. 40.5%), those aged under 35 years compared with respondents aged 35 or over (48.4% vs. 43.6%) those with other children in the family in addition to the 4-year-old child (46.8% vs. 40.5%), and respondents in a single-parent family compared to those with two adults in the family (51.3% vs. 45.3%).

**Table 2.** Associations Between FV and Background Variables and Risk Factors, %, Cross-Tabulations, Chi-Square Tests, and Cramer’s *V* Tests.

	Parents Who Reported FV^a^ (*n* = 10,694)			
	Yes	No	*p*-value	Cramer’s *V*-value^b^
Background Variables	*n* (%)	*n* (%)		
All parents	4,890 (45.7)	5,804 (54.3)		
Parents’ background variables				
Gender			<.001	.07
Female (*n* = 7,619)	3,637 (47.7)	3,982 (52.3)		
Male (*n* = 3,023)	1,223 (40.5)	1,800 (59.5)		
Age			<.001	.05
<35 years old (*n* = 4,792)	2,319 (48.4)	2,473 (51.6)		
≥35 years old (*n* = 5,879)	2,561 (43.6)	3,318 (56.4)		
Number of children in the family			<.001	.05
4-year-old is only child (*n* = 1,708)	691 (40.5)	1,017 (59.5)		
Other children in the family (*n* = 8,849)	4,137 (46.8)	4,712 (53.2)		
Number of adults in the family			.001	.03
One adult in the family (*n* = 782)	401 (51.3)	381 (48.7)		
Two adults in the family (*n* = 9,859)	4,466 (45.3)	5,393 (54.7)		
Parent-related risk factors for family violence				
Parent’s recent situation				
Has a chronic illness or other chronic health problem (*n* = 10,642)			<.001	.04
Yes	1,386 (49.2)	1,432 (50.8)		
No	3,480 (44.5)	4,344 (55.5)		
Perceived health condition at most average (*n* = 10,644)			<.001	.07
Yes	578 (56.8)	440 (43.2)		
No	4,287 (44.5)	5,339 (55.5)		
Has restrictions caused by a health problem (*n* = 10,628)			<.001	.08
Yes	1,008 (54.2)	852 (45.8)		
No	3,860 (44.0)	4,908 (56.0)		
Felt of loneliness fairly often or all the time (*n* = 10,671)			<.001	.12
Yes	494 (68.0)	233 (32.0)		
No	4,384 (44.1)	5,560 (55.9)		
Depressive symptoms for at least a period of 2 weeks over the past 12 months (*n* = 10,605)			<.001	.17
Yes	1,276 (62.7)	758 (37.3)		
No	3,572 (41.7)	4,999 (58.3)		
Psychological distress over the past 4 weeks (*n* = 10,513)			<.001	.13
Yes	467 (72.1)	181 (27.9)		
No	4,362 (44.2)	5,503 (55.8)		
Been worried about coping as a parent often or always (*n* = 10,664)			<.001	.15
Yes	505 (73.8)	179 (26.2)		
No	4,370 (43.8)	5,610 (56.2)		
Felt inadequate as a parent often or always (*n* = 10,640)			<.001	.18
Yes	898 (70.6)	374 (29.4)		
No	3,972 (42.4)	5,396 (57.6)		
Parent’s childhood adversities				
Long-term financial difficulties in the family (*n* = 10,650)			<.001	.07
Yes	1,918 (50.6)	1,871 (49.4)		
No	2,950 (43.0)	3,911 (57.0)		
Parents’ divorce (*n* = 10,650)			.924	.00
Yes	1,196 (45.8)	1,417 (54.2)		
No	3,670 (45.7)	4,367 (54.3)		
Parents’ regular unemployment (*n* = 10,656)			.199	.01
Yes	836 (47.1)	939 (52.9)		
No	4,035 (45.4)	4,846 (54.6)		
Bullying at school (*n* = 10,653)			<.001	.09
Yes	1,925 (51.6)	1,804 (48.4)		
No	2,940 (42.5)	3,984 (57.5)		
Serious conflicts within the family (*n* = 10,647)			<.001	.10
Yes	1,605 (53.9)	1,374 (46.1)		
No	3,261 (42.5)	4,407 (57.5)		
Parent’s mental health problems (*n* = 10,669)			<.001	.07
Yes	853 (54.1)	723 (45.9)		
No	4,023 (44.2)	5,070 (55.8)		
Parent’s alcohol problems (*n* = 10,670)			<.001	.05
Yes	1,310 (49.6)	1,329 (50.4)		
No	3,567 (44.4)	4,464 (55.6)		

*Note.* FV = family violence.

a Sum variable.

b The degrees of freedom = 1 in all analyses.

Compared to the parents reporting no violence, reporting FV was more frequent among those parents with a chronic illness or other health problem (49.2% vs. 44.5%), those who rated their health as average at best (56.8% vs. 44.5%), those who felt restricted by their health problem (54.2% vs. 44.0%), those feeling lonely often or all the time (68.0% vs. 44.1%), those with depressive symptoms for at least a period of 2 weeks over the past 12 months (62.7% vs. 41.7%), those experiencing psychological distress over the past 4 weeks (72.1% vs. 44.2%), feeling often or always worried about their coping as a parent (73.8% vs. 43.8%), and often or always feeling inadequate as a parent (70.6% vs. 42.4%).

In the context of the parents’ childhood adversities, reporting FV was more frequent among those parents who reported having faced long-term financial difficulties in their childhood family (50.6% vs. 43.0%), being subject to bullying at school (51.6% vs. 42.5%), experiencing serious conflicts in their childhood family (53.9% vs. 42.5%), and who reported their parents’ mental health problems (54.1% vs. 44.2%) alcohol problems (49.6% vs. 44.5%) in the childhood family compared to those parents who reported no childhood adversities. As shown in Table 2, there were no associations between FV and parents’ divorce (*p* = .924) and parents’ regular unemployment (*p* = .199). Therefore, these two variables were also excluded from further analyses. All other associations were statistically significant, but effect sizes were small.

### Parent-Related Risk Factor Clusters by EFA

Table 3 shows how parent-related risk factors for FV load on three factors. The factor model proved to have significant consistency content and was found to be statistically acceptable. In this article, the content of the factor model is divided into three clusters referred to as the “risk factor clusters.” The first risk factor cluster, *parental well-being*, included five parent-related risk factors: feelings of loneliness, depressive symptoms, psychological distress, being worried about coping as a parent, and feeling inadequate as a parent. The second risk factor cluster, *parent’s childhood adversities*, included five parent-related risk factors: long-term financial difficulties, bullying at school, serious conflicts in the family, the parent’s mental health problems, and the parent’s alcohol problem. The third risk factor cluster, *parent’s health*, included three parent-related risk factors: the parent has a chronic illness or other chronic health problem, self-rated health is at most at an average level, and experiences restrictions due to a health problem (Table 3).

**Table 3.** Factor Model and Communalities and Factor Loadings of the Parent-Related Risk Factors by Exploratory Factor Analysis.

	Commu-nalities	Factor Loadings		
Parent-Related Risk Factor Clusters		F1	F2	F3
Risk factor cluster 1: Parental well-being				
Felt lonely fairly often or all the time	0.32	0.54		
Depressive symptoms for at least a period of 2 weeks over the past 12 months	0.36	0.56		
Psychological distress over the past 4 weeks	0.49	0.68		
Been worried about coping as a parent often or always	0.57	0.76		
Felt inadequate as a parent often or always	0.50	0.71		
Risk factor cluster 2: Parent’s childhood adversities				
Long-term financial difficulties within the family	0.40		0.62	
Bullying at school	0.15		0.32	
Serious conflicts within the family	0.59		0.76	
Parent’s mental health problems	0.38		0.61	
Parent’s alcohol problems	0.51		0.71	
Risk factor cluster 3: Parent’s health				
Has a chronic illness or other chronic health problem	0.65			0.80
Perceived health condition at most average	0.48			0.61
Has restrictions caused by a health problem	0.69			0.81

Extraction method: principal component analysis.

Rotation: Varimax.

Communality values varied acceptably between 0.32 and 0.69 except for bullying at school (0.15). Similarly, factor loadings varied acceptably between 0.54 and 0.81 except for bullying at school (0.32). However, bullying at school was considered such a meaningful risk factor (e.g., Aromaa & Koskinen, 2002) that it was included in the parent’s childhood adversities factor (Table 3).

#### Occurrence of FV According to the Accumulation of Risk Factors

Table 4 shows the numbers and percentages of the risk factors with the three risk factor clusters for FV (yes/no). Of those parents who reported FV, 59.4% did not have any risk factors in the parental well-being cluster, 28.4% did not have any risk factors in the parent’s childhood adversities cluster, and 62.6% did not have any risk factors in the parent’s health cluster. The corresponding figures for those parents who did not report FV were 80.5%, 38.9%, and 69.8%. Nearly three-quarters of those parents (71.6%) who reported FV had at least one risk factor in the parent’s childhood adversities cluster, while the corresponding figures were 40.6% for the parental well-being cluster and 37.4% for the parent’s health cluster (Table 4).

**Table 4.** Numbers and Percentages of the Risk Factors with the Three Risk Clusters for FV.

	Parental Well-being Cluster		Parent’s Childhood Adversities Cluster		Parent’s Health Cluster	
	Parents Exposed to FV		Parents Exposed to FV		Parents Exposed to FV	
Number of Risk Factors	Yes	No	Yes	No	Yes	No
	*n* (%)	*n* (%)	*n* (%)	*n* (%)	*n* (%)	*n* (%)
None	2,904 (59.4)	4,672 (80.5)	1,385 (28.4)	2,252 (38.9)	3,057 (62.6)	4,046 (69.8)
1	1,073 (21.9)	764 (13.2)	1,318 (27.0)	1,574 (27.1)	985 (20.2)	1,017 (17.5)
2	456 (9.3)	214 (3.7)	940 (19.3)	922 (15.9)	542 (11.0)	501 (8.6)
3	236 (4.8)	94 (1.6)	672 (13.8)	607 (10.5)	301 (6.2)	235 (4.1)
4–5	220 (4.5)	59 (1.0)	562 (11.5)	438 (7.6)		
Total^a^	4,889 (100)	4,877 (100)	4,977 (100)	5,793 (100)	4,885 (100)	5,799 (100)

*Note.* FV = family violence.

a The same parent had been able to answer more than one option.

The following paragraphs include a more detailed look at how a number of risk factors may predict the occurrence of FV. In general, in all three clusters without any risk factors, the prevalence of FV is lower when compared to total respondents (38.1%–43.0% vs. 45.7%). Adjusting the background factors did not significantly affect the results: the more risk factors affected the parents’ well-being, the more likely they were to also report FV (Table 5).

**Table 5.** Occurrence of Family Violence According to the Accumulation of Risk Factors Within the Risk Factor Cluster, by Cross-Tabulations (*n*, %), Chi-Square Tests and Cramer’s *V* Tests and Logistic Regression Analyses (OR, 95% Confidence Intervals).

Number of Risk Factors	Occurrence of Family Violence According to the Number of Risk Factors Within the Risk Factor Cluster											
	Parental Well-being Cluster				Parent’s Childhood Adversities Cluster				Parent’s Health Cluster			
	*n* (%)	Cramer’s *V* (*df* = 4)	OR	95% CI	*n* (%)	Cramer’s *V* (*df* = 4)	OR	95% CI	*n* (%)	Cramer’s *V* (*df* = 3)	OR	95% CI
		.24				.13				.08		
None	2,904 (38.3)		1		1,385 (38.1)		1		3,057 (43.0)		1	
1	1,073 (58.4)		2.21	1.99, 2.45	1,318 (45.6)		1.35	1.22, 1.50	985 (49.2)		1.26	1.14, 1.39
2	456 (68.1)		3.38	2.85, 4.02	940 (50.5)		1.61	1.43, 1.80	542 (52.0)		1.40	1.23, 1.60
3	236 (71.5)		3.97	3.10, 5.09	672 (52.5)		1.79	1.57, 2.04	301 (56.2)		1.71	1.43, 2.05
4–5	220 (78.9)		6.05	4.48, 8.18	562 (56.2)		2.03	1.75, 2.34				

Regression analysis adjusted for parent’s gender, age, number of children in the family, and number of adults in the family.

All associations were statistically very significant, *p*-values < .001.

##### Risk Factor Cluster 1: Parental Well-being as a Predictor of FV

Table 5 shows that of those parents unaffected by the well-being risk factors in risk factor cluster 1, 38.3% reported FV. Of those parents affected by a single risk factor, 58.4% reported FV. In other words, one risk factor alone increases the prevalence of FV compared to all respondents (58.4% vs. 45.7%). Of the parents with two risk factors, 68.1% reported FV, and 71.5% reported FV if there were three risk factors. Of the parents with at least four risk factors, 78.9% of reported FV. The effect size (*V* = .24, *df* = 4) may be interpreted as large. In comparison with the parents with no risk factors, the likelihood of the occurrence of FV was twice as high in parents with one well-being risk factor (OR = 2.21; CI: [1.99, 2.45]) and six times as high in parents with between four and five risk factors (OR = 6.05; CI: [4.48, 8.18]) (Table 5).

##### Risk Factor Cluster 2: Childhood Adversities as a Predictor of FV

When a parent did not have any risk factors within the childhood adversities risk factor cluster, the occurrence of FV was 38.1%. Of those parents who had a single risk factor, 45.6% reported FV. In other words, the incidence of a single risk factor for FV was the same for them as for all respondents (45.6% vs. 45.7%). Of the parents with two risk factors, 50.5% reported FV; of those with three risk factors, 52.5% reported FV. Of the parents with at least four risk factors, 56.2% reported FV. The effect size (*V* = .13, *df* = 4) may be interpreted as medium. The odds ratio for the occurrence of FV in parents with one childhood adversity risk factor was 1.35 (CI: [1.22, 1.50]). With at least four risk factors, the odds for FV were double (OR = 2.03; CI: [1.75, 2.34]), which is almost the same as for those with one risk factor in the parental well-being cluster (2.21) (Table 5).

##### Risk Factor Cluster 3: Parent’s Health as a Predictor of FV

For the parents without any risk factors within the parent’s health risk factor cluster, the occurrence of FV was 43.0%. Of those parents with a single risk factor, 49.2% reported FV. In other words, the incidence of FV among those with one risk factor is slightly higher compared to all respondents (49.2% vs. 45.7%). Of the parents with two risk factors, 52.0% reported FV. Of the parents with three reported risk factors (maximum for this cluster), 56.2% reported FV. The effect size (*V* = .08, *df* = 3) may be interpreted as small. The odds ratio for the occurrence of FV in parents with one health risk factor was 1.26 (CI: [1.14, 1.39]). With three risk factors, the odds ratio for FV was 1.71 (CI: [1.43, 2.05]). This figure is nearly the same as for those with one risk factor in the parental well-being cluster (2.21), and at least four risk factors in the parent’s childhood adversities cluster (2.03) (Table 5).

## Discussion

Based on our data, IPV and CM were found to be relatively common. About one in ten parents, including both men and women, reported experiencing some form of IPV. Furthermore, according to the parents’ reports, less than half of 4-year-olds have been exposed to some form of CM at least once during the previous 12 months. In this study, we did not distinguish between the experiences of violence among men and women or the different forms of CM and IPV that may occur in the families. As a result, it is not possible to make direct comparisons with previous studies on the prevalence of FV based on gender.

Based on earlier evidence, we know that IPV is a well-known risk factor for CM (e.g., Brown et al., 2021; Leppäkoki et al., 2021; Younas & Morrison Gutman, 2022) and that these may co-occur (e.g., Birarra et al., 2016; van Berkel et al., 2020; Leppäkoski et al., 2021). The results of the present study indicated that some forms of CM may have co-occurred in the context of IPV because some parents reported both IPV and CM. The accumulation of acts of violence with life-long health consequences is a serious problem. It is likely that some 4-year-olds and their parents have experienced recurrent FV, although the exact share of those affected remains unclear in this study. As experiences of violence affect individuals’ health and well-being throughout their life span, the social and health professionals providing families with services should ask about FV whenever there is any suspicion of this and even when there is no actual concern. There is strong evidence that many factors such as maternal depression, maternal history of childhood maltreatment, or marital distress may increase the risk of CM (e.g., Younas & Morrison Gutman, 2022). However, they did not emerge in the present study. It is important to notice that an individual risk factor does not as such indicate CM. The assessment of the conditions in the family that may increase the risk for CM must always attention to the family’s situation as a whole (Rantanen et al., 2022, p. 3).

Effect size helps readers understand the magnitude of differences found, whereas statistical significance examines whether the findings are likely to be due to chance increasing the power of the present study (Sullivan & Feinn, 2012). Several of the parent-related risk factors detected in this study were associated with the likelihood of FV. Our findings indicate that the following individual risk factors were associated with an increased risk for FV: female gender, parent’s age under 35 years, other children in the family, and a single-parent family. Similar results have been obtained by van Berkel et al. (2020) and Peltonen et al. (2014), among others. Furthermore, our research results indicated a significant association between FV and several risk factors describing the parent’s recent situation, such as experiencing depressive symptoms, psychological distress, being worried about coping as a parent, and feeling inadequate as a parent. The research results show that factors related to the parent’s well-being seem to be much more strongly associated with the risk of FV than background factors. For example, family-related stress (Peltonen et al., 2014) or parental stress (Liel et al., 2020; Younas & Morrison Gutman, 2022) have been detected as risk factors for various forms of CM. In this respect, the results of our study are in line with previous research findings. Thus, our research results can be considered trustworthy and usable.

As regards the childhood adversities faced by the parent respondents, a quarter of the parents of 4-year-olds reported having experienced their parents’ divorce and almost a sixth of the respondents had experienced their parents’ long-term unemployment during their childhood. Nevertheless, our study indicated that these factors were not associated with an increased risk of FV as individual risk factors. However, our results showed that nearly three-quarters of those parents who had been subjected to FV reported at least one childhood adversity, including long-term financial difficulties or bullying at school, serious conflicts in the family, or their parent’s mental health or alcohol problems. This result is in line with a previous study (Chartier et al., 2010), in which 72% of a representative population sample reported at least one adverse childhood experience (e.g., CM, parental marital conflict, poor parent–child relationship, and parental mental health issues). Furthermore, our research results indicated that the accumulation of childhood adversities significantly increased the risk of FV. Furthermore, there is evidence-based research identifying associations between ACEs and poor health later in life (e.g., Bellis et al., 2019; Mosley-Johnson et al., 2019), and multiple ACEs have also been found to be related to ACE risks for the following generation (Hughes et al., 2017).

As it is now recognized that no single risk factor alone is necessarily predictive of family violence, we wanted to look at whether cross-tabulations would reveal any risk factors associated with FV related to one another also through some latent feature. In other words, we wanted to find out whether there are hidden similarities, that is, clusters, among the risk factors. We found that risk factors clearly loaded onto three clusters, supporting the idea that these risk factors could be viewed as groups. As a result, we found an answer to our question about what kinds of parents report FV. In addition, it is possible to consider the benefits of such a cluster analysis instead of merely focusing on examining individual risk factors. We found that the accumulation of risk factors across all clusters increases the likelihood of FV. The accumulation of risk factors has a particularly strong impact on the well-being cluster.

Our findings suggest that family services should pay attention to factors that impair the well-being and health of parents. Any individual concerns raised by parents should be addressed by those who encounter families at their work or through their social networks (see also Bekaert et al., 2021). Social support has been found to be the most significant protective factor against CM (Younas & Morrison Gutman, 2022). Higher availability of services (e.g., social services), community involvement, and support from friends and family can all provide protection against CM. For instance, this can mean conversations as an intervention method between a nurse and a child’s parent (Bekaert et al., 2021; Lepistö et al., 2022) in addition to a higher availability of services (Younas & Morrison Gutman, 2022). Moreover, special attention must be paid to the coping and survival of parents in their parenthood role. For example, Peltonen et al. (2014) showed that mothers considered a lack of help for dealing with parenting problems as a risk factor for severe acts of violence toward their children in addition to family-related stress. It is likely that when risks increase (≥5), families will not be able to cope adequately without help, and the likelihood of different forms of FV increases, as also argued by Yang and Maguire-Jack (2018) in the case of CM.

## Limitations

The current study has several limitations. The use of self-reported data and a retrospective study design may limit the validity of the measures used. Furthermore, parents may remember things incorrectly or, due to the sensitivity of the questions, may omit some facts because FV is still a sensitive matter. However, self-reporting, the method used in the present study, has been found more reliable than evaluations carried out by an outsider (Rantanen et al., 2022).

Furthermore, the scope of this study is limited, as the survey of which some data were used in the present study was not originally designed for the specific purpose of investigating FV. Instead, the experiences of FV were only a small part of a broader welfare study. On the positive side, however, this study utilized a fairly large and comprehensive data set. The scope of the data enabled extensive examination of the connections between different issues.

## Conclusions

Our findings emphasize the importance of preventing any forms of FV as early as possible. We can conclude on a general level that the more risk factors affected the parents, the more likely they were to report FV. The parents examined in the present study expressed many worries and faced many challenges. Even a single risk factor was highly likely to predict an increased risk for FV. We emphasize that individual concerns and worries must also be taken into account in discussions with parents, especially by paying attention to the well-being of parents. Parents should be systemically asked about any possible risk factors. The accumulation of risk factors for well-being has the biggest impact on the risk of FV. A family exposed to several risk factors will not be able to cope adequately without help, and the parents may not have the strength to ask for help themselves. Help should be provided in cooperation with the parents by directly addressing any relevant issues. Further research should investigate the need and adequacy of family services and the support and help given to and received by the parents of 4-year-old children. Furthermore, in our opinion, the factor analysis worked well both in terms of content and technical aspects. It also produced reasonable results. Our research results showed that factors related to parents’ personal well-being are more significant risk factors than the adversities they have experienced during their childhood or the parents’ health. This is useful information when assessing the risks of FV (IPV and/or CM).
